# Sympatric Occurrence of *Rickettsia slovaca* and *Rickettsia raoultii* in *Dermacentor* Ticks from Samara Oblast and the First Molecular Detection of *Rickettsia felis* in Russia and Globally in *Dermacentor reticulatus*

**DOI:** 10.3390/microorganisms14071461

**Published:** 2026-07-02

**Authors:** Alexey V. Rakov, Tatiana A. Chekanova, Ketevan Petremgvdlishvili, Tatiana V. Vandysheva, Nikita I. Myasnikov, Alina M. Devyatova, Anna A. Ukhvakova, Vasiliy G. Akimkin

**Affiliations:** 1 Laboratory for Natural Focal Infections Epidemiology, Central Research Institute of Epidemiology, 111123 Moscow, Russia; tchekanova74@mail.ru (T.A.C.); ketevan0511@mail.ru (K.P.); miasnikov@cmd.su (N.I.M.); 2 Center of Hygiene and Epidemiology in the Samara Region, 443079 Samara, Russia; vandyshevatv@fguzsamo.ru (T.V.V.); zemit13@mail.ru (A.M.D.); yhvakovaaa@fguzsamo.ru (A.A.U.); 3 Central Research Institute of Epidemiology, 111123 Moscow, Russia; vgakimkin@yandex.ru

**Keywords:** *Rickettsia*, *Rickettsia felis*, *Rickettsia slovaca*, *Rickettsia raoultii*, SFGR, sympatry, *Dermacentor*, Samara, Russia

## Abstract

Samara Oblast is an ecological transition zone in Russia, yet its spotted fever group *Rickettsia* (SFGR) diversity and prevalence in *Dermacentor* ticks remain unexplored. This study characterized SFGR species circulating in *Dermacentor reticulatus* and *Dermacentor marginatus* across 15 districts of Samara Oblast. We collected 681 adult *Dermacentor* ticks via vegetation flagging during 2023–2025. SFGR screening was performed via a commercial qPCR kit. Genospecies were identified via Sanger sequencing of the partial *gltA* gene and validated by means of *ompB* analysis, while tick species were confirmed using a *cox1* gene fragment. The overall SFGR prevalence was 33.5% (228/681). Infection rates were significantly higher in *D. marginatus* (44.7%) than in *D. reticulatus* (27.5%). *Rickettsia raoultii* predominated (94.4%), while *Rickettsia slovaca* was relatively rare (4.6%) and significantly associated with *D. marginatus*. Notably, partial *ompB* sequencing revealed two distinct *R. raoultii* putative genotypes circulating in the region: one with a 9 bp deletion and one without. No coinfections were detected. Unexpectedly, a single *D. reticulatus* tick tested positive for *Rickettsia felis*, confirmed by means of *gltA* and *ompB*, marking the first molecular detection of *R. felis* in Russia and globally in this tick species. Samara Oblast represents a high-prevalence sympatric zone for *Dermacentor*-borne SFGR. The molecular detection of *R. felis* in *D. reticulatus* suggests a potential association that requires confirmation through further studies.

## 1. Introduction

The spotted fever group *Rickettsia* (SFGR) is a diverse group of obligate intracellular Gram-negative bacteria that can cause various tick-borne diseases (TBDs). Ixodid ticks (Acari: Ixodidae) serve as the primary vectors for SFGR and other important tick-borne pathogens (TBPs). In Europe, the two most common ixodid tick genera are *Ixodes* and *Dermacentor*. *Ixodes ricinus* (Linnaeus, 1758), commonly known as the woodland tick, is the most widespread species, mainly inhabiting forest ecosystems with dense vegetation and leaf litter [1]. It is the main reservoir of the causative agents of Lyme disease and tick-borne encephalitis. *Dermacentor reticulatus* (Fabricius, 1794), the meadow tick, and *D. marginatus* (Sulzer, 1776), the ornate sheep tick, are known vectors of *Rickettsia conorii* subsp. *raoultii* (basonym: *R. raoultii*) and *Rickettsia slovaca*, both widely distributed across Europe [2]. *D. marginatus* predominantly occurs in Southern Europe, particularly in regions with dense shrub and forested areas, where oak and pine trees are common. In contrast, *D. reticulatus* is frequently found across the colder northern areas of Western Europe and the territories of the former Soviet Union, characterized by high humidity and relatively mild winters. Both tick species, *D. marginatus* and *D. reticulatus*, are considered reservoirs for *R. raoultii* and *R. slovaca*, which persist in tick populations through transstadial and transovarial transmission. Consequently, the geographic distribution of these rickettsiae mirrors that of *Dermacentor* hosts [3].

Regarding the prevalence of one of the two SFGR species in *Dermacentor* spp., there are some controversial opinions. For example, according to one study, *D. reticulatus* ticks were more infected with *R. raoultii*, whereas *D. marginatus* shows higher infection rates with *R. slovaca* [4]. In other reports, *R. raoultii* seems to be more highly prevalent in both *D. marginatus* and *D. reticulatus* populations in nature than *R. slovaca* [3].

Both SFGR species—*R. slovaca* and later *R. raoultii*—have been described as causative agents of TIBOLA/DEBONEL/SENLAT syndrome (also known as tick-borne lymphadenopathy) characterized by a scalp eschar and cervical lymphadenopathy, a disease that is quite difficult to diagnose due to the lack of pathognomonic symptoms. Tick-borne lymphadenopathy is the most common tick-borne rickettsiosis in Europe after Mediterranean spotted fever (MSF) [5]. It has been suggested that *R. slovaca* is more significantly associated with TIBOLA/DEBONEL/SENLAT patients than *R. raoultii* (*p* < 0.05). More cases of *R. slovaca* infection have been recorded, which suggests that *R. raoultii* is less pathogenic. However, TIBOLA/DEBONEL/SENLAT is a relatively recently recognized disease, and its incidence is likely underestimated [3]. Overall, TIBOLA can be considered as a relatively rare and mild tick-borne human infection compared to Lyme borreliosis [6].

Russia remains insufficiently studied in terms of the distribution of various TBPs and their species across its vast territory, as well as the challenges in diagnosing rickettsioses other than North Asian tick typhus (NATT) and Astrakhan spotted fever (ASF), which are monitored by sanitary and epidemiological surveillance authorities. In Russia, *R. raoultii* and *R. slovaca* are known to circulate within *Dermacentor* species [7,8,9,10]. The ability of these species to occupy the same ecological niche raises important questions about interspecific interactions, co-transmission dynamics, and the potential emergence of novel rickettsial species.

To date, no information has been available on the prevalence and species diversity of SFGR in *Dermacentor* ticks in Samara Oblast in the Volga Federal District, Russia. We can only infer the information to compare from neighboring regions, including West Kazakhstan. Samara Oblast is a strategically important region of Russia, a key industrial, transport, and space center of the Volga region.

The primary objective of this study was to characterize the prevalence, species diversity, and distribution patterns of SFGR, specifically *R. slovaca* and *R. raoultii*, within sympatric populations of *D. reticulatus* and *D. marginatus* across 15 districts of the Samara Oblast. Occasionally, we have found novel to Russia *Rickettsia felis*, a species mostly associated with cat fleas, in *D. reticulatus*, which had not been previously detected in this tick species globally.

## 2. Materials and Methods

### 2.1. Study Area and Tick Collection

**Figure 1:**
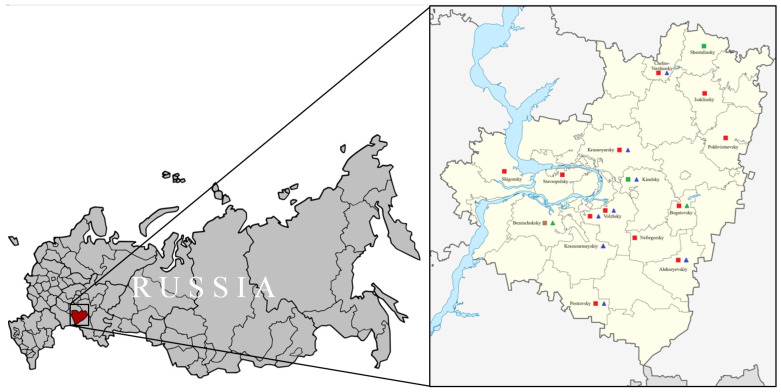
Map of tick collection sites in the Samara Oblast. The collection sites are marked with squares and triangles for *D. reticulatus* and *D. marginatus*, respectively. Color code: Red—*R. raoultii* in *D. reticulatus*, Blue—*R. raoultii* in *D. marginatus*, Green—*R. raoultii* + *R. slovaca*, Brown—*R. raoultii* + *R. felis*. Modified from https://upload.wikimedia.org/wikipedia/commons/5/53/Outline_Map_of_Samara_Oblast.svg; accessed on 29 May 2026.

The Samara Oblast of the Russian Federation borders Tatarstan in the north, Orenburg Oblast in the east, Kazakhstan (West Kazakhstan Region) in the south, Saratov Oblast in the southwest, and Ulyanovsk Oblast in the west (Figure 1).

The Samara Oblast is characterized by a markedly continental climate, with a mean annual air temperature of +3.8 °C. The average temperature in January is −13.9 °C, while in July it reaches +20.1 °C. The region lies within the forest-steppe zone: its northern part is predominantly covered by pine and mixed broad-leaved forests, whereas the southern and eastern territories are largely occupied by steppe ecosystems.

Tick samples were collected from vegetation using the flagging method during April and May over a three-year period (2023–2025), covering 15 of the 27 administrative districts of the Samara Oblast (55.6%) (Figure 1). For each sampling site, geographic coordinates, tick species, and the number of collected samples were recorded. All collected questing ticks were unfed (starved). Ticks attached to the flag were removed with tweezers, placed into individual Eppendorf tubes and transported to the laboratory. Upon arrival, they were stored at −20 °C until further processing and identification. In addition, a total of 225 *Dermacentor* spp. ticks, grouped in pools, were collected in May 2022 from 3 districts as part of a pilot study to assess SFGR species composition. Morphological tick species identification was conducted using standard taxonomic keys [11].

### 2.2. DNA Extraction and Quantitative PCR

Each tick was individually washed with 96% ethanol and then 0.15 M sodium chloride solution. Ticks were homogenized in a 2.0 mL Eppendorf tube containing 300 μL of 0.15 M sodium chloride with tungsten carbide beads in a TissueLyser LT homogenizer (Qiagen, Hilden, Germany) at 50 Hz for 10 min. The total DNA was extracted from 100 μL of tick lysate using the AmpliSens^®^ Ribo-PREP kit (CRIE, Moscow, Russia). DNA extracts were stored at −20 °C until further analysis. Detection of SFGR in adult *Dermacentor* ticks was performed via quantitative polymerase chain reaction (qPCR) with the AmpliSens^®^ *Rickettsia* spp. SFG-FL kit (CRIE, Moscow, Russia) targeting the *ompB* gene on the RotorGene Q real-time PCR cycler (Qiagen, Hilden, Germany). We also used the next kits manufactured by CRIE, Moscow, Russia for detection of: *Coxiella burnetii* (using the AmpliSens^®^ *Coxiella burnetii*-FL kit), *Borrelia miyamotoi* (using the AmpliSens^®^ *Borrelia miyamotoi*-FL kit targeting the *glpQ* gene), TBEV, *Borrelia burgdorferi* s.l., *Anaplasma phagocytophilum*, and *Ehrlichia chaffeensis*/*Ehrlichia muris* (using the AmpliSens^®^ TBEV, *B. burgdorferi* sl, *A. phagocytophilum*, *E. chaffeensis/E. muris*-FL kit targeting *C*, 16S rRNA, *msp2*, and 16S rRNA genes, respectively). All procedures were performed according to the manufacturers’ instructions.

### 2.3. Conventional PCR and Sanger Sequencing

The SFGR genospecies were determined via Sanger sequencing of the pan-rickettsial citrate synthase *gltA* (384 bp) partial gene on both DNA strands using specific primers, as described previously [7]. Additionally, primers targeting gene fragments of outer membrane protein A (*ompA*, 532 bp), outer membrane protein B (*ompB*, 426 bp), 17-kDa protein (*htrA*, 450 bp), 16S rRNA (*rrs*, 772 bp), and heat-stable 120-kDa protein (*sca4*, 844 bp) were used for *R. felis* determination [7,12].

For confirmation of tick species in DNA samples, conventional PCR followed by Sanger sequencing was performed, targeting a 710 bp fragment of the mitochondrial cytochrome c oxidase subunit I (*cox1* or *COI*) gene in invertebrates. The primers used were LCO1490 (5′-GGT CAA CAA ATC ATA AAG ATA TTG G-3′) and HCO2198 (5′-TAA ACT TCA GGG TGA CCA AAA AAT CA-3′) [13]. The thermal profile consisted of an initial denaturation at 95 °C for 5 min; 40 cycles of denaturation at 94 °C for 40 s, annealing at 45 °C for 60 s, and extension at 72 °C for 60 s; followed by a final extension at 72 °C for 10 min. Homologous sequences were identified in the GenBank nr/nt database using BLASTN 2.17.0 with the default parameters.

### 2.4. Phylogenetic and Statistical Analysis

Dendrograms were constructed in MEGA 6.06 software with the maximum likelihood method on aligned sequences of both genes, with 1000 bootstrap replicates. For comparison, homologous sequences from complete genomes of representative SFGR available in GenBank were used. Homologous fragments from the “ancestral group” species *Rickettsia bellii* An04 (NZ_CP015010) and *Rickettsia canadensis* CA410 (NC_016929) genome sequences were used as outgroups to construct dendrograms based on the partial *gltA* and *ompB* gene sequences, respectively.

For the tick infection rates, 95% confidence intervals (CIs) and the two-sample *z*-test to compare sample proportions were calculated using Epitools (https://epitools.ausvet.com.au (accessed on 26 May 2026)) [14]. A *p*-value ≤ 0.05 was considered statistically significant.

The partial *gltA*, *ompB*, and *cox1* gene sequences obtained in this study have been deposited in GenBank (Acc. No. PZ166815-PZ166934, PZ436268-PZ436272, PZ448574-PZ448579).

## 3. Results

### 3.1. Prevalence of Tick-Borne Pathogens in Dermacentor Ticks in Samara Oblast

Morphological identification of the collected *Dermacentor* ticks confirmed that Samara Oblast is a sympatric area for both *D. reticulatus* and *D. marginatus*, as both species were collected simultaneously at the same sampling sites (Table 1). A total of 444 *D. reticulatus* (65.2%) and 237 *D. marginatus* (34.8%) ticks were collected. Both species co-occurred in eight of the 15 surveyed districts; six districts yielded only *D. reticulatus*, while *D. marginatus* was the sole species found in one district (Figure 1).

**Figure 2:**
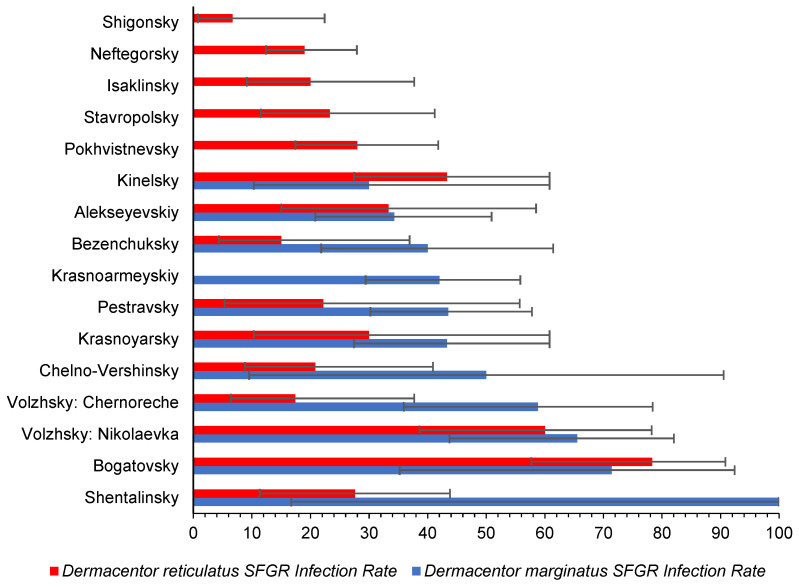
SFGR prevalence (%) with 95% confidence intervals in *D. reticulatus* (red bars) and *D. marginatus* (blue bars) across the studied districts of Samara Oblast, 2023–2025.

Overall, 228 of the 681 collected *Dermacentor* (33.5%, 95% CI: 30.0–37.1%) tested positive for SFGR (Table 1). This distribution summarizes the prevalence of SFGR in *Dermacentor* ticks during 2023–2025 and shows a significantly higher prevalence in *D. marginatus* (44.7%, 95% CI: 38.5–51.1%) than in *D. reticulatus* (27.5%, 95% CI: 23.5–31.8%) (*p* < 0.0001). Among the SFGR-positive ticks, 47.4% (*n* = 108) were successfully sequenced to determine the genospecies composition of SFGR. Detailed district-level prevalence data are provided in Table 2. The highest infection rates were recorded in the Bogatovsky District, with 78.3% (95% CI: 57.7–90.8%) in *D. reticulatus* and 71.4% (95% CI: 35.2–92.4%) in *D. marginatus*. In contrast, the lowest SFGR prevalence in *D. reticulatus* was observed in Bezenchuksky (15.0%; 95% CI: 4.4–36.9%) and Volzhsky (17.4%; 95% CI: 6.4–37.7%) Districts (Figure 2). SFGR was detected in at least some ticks across all studied districts.

TBPs other than SFGR (TBEV, *B. burgdorferi* s.l., *B. miyamotoi*, *A. phagocytophilum*, *E. chaffeensis*, *E. muris*, and *C. burnetii*) were not detected in the tick samples using the corresponding qPCR kits.

### 3.2. SFGR Genospecies and Their Sympatry Observed in Dermacentor Ticks in Samara Oblast

Supplementary Table S1 presents results from the 2022 preliminary pilot study in three districts, which identified *R. slovaca* in Kinelsky and Krasnoyarsky Districts and *R. raoultii* in two sites within the Volzhsky District. Additionally, in the same year, *R. raoultii* was identified in an SFGR-positive tick removed from a patient.

**Figure 3:**
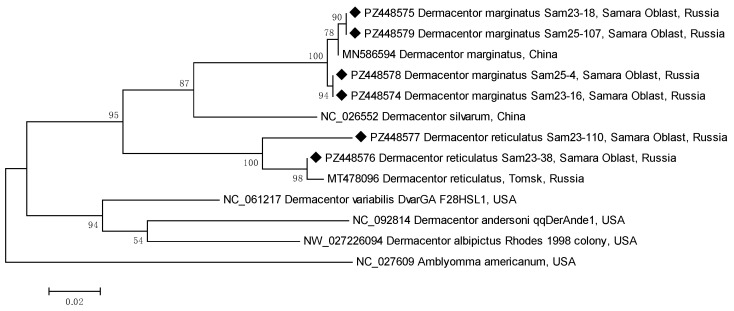
Phylogenetic tree constructed using the maximum likelihood method based on nucleotide sequences of *Dermacentor* spp. ticks, including six from this study (Samara Oblast, black diamonds, ♦) and reference sequences of the *cox1* gene fragment (710 bp). The *Amblyomma americanum* (NC_027609) sequence was used as an outgroup. The GenBank accession numbers for reference sequences are shown with the sequence name, tick species, and country. The branch numbers indicate bootstrap support (1000 replicates). The scale bar indicates phylogenetic distance.

To assess the prevalence of specific SFGR species, 71 randomly selected SFGR-positive samples (up to 3 ticks per district) were sequenced using the partial *gltA* gene. In the four districts where *R. slovaca* was detected, all remaining samples from those districts were sequenced to fully determine the species’ prevalence. In total, 108 of the 228 SFGR-positive ticks (47.4%) were sequenced (Table 2). Of the 108 identified samples, *R. raoultii* was significantly more prevalent (102; 94.4%, 95% CI: 90.1–98.8%) than *R. slovaca* (5; 4.6%, 95% CI: 0.7–8.6%) (*p* < 0.0001). Overall, only three of 15 districts had a single *R. slovaca* sample (Kinelsky, Bogatovsky, and Shentalinsky Districts), while two samples were found in Bezenchuksky District (18.2%, 95% CI: 0.0–41.0%). To confirm the correct morphological identification of tick species, we performed sequencing of the *cox1* gene fragment of all five *R. slovaca*-positive samples (Figure 3). Four of the five *R. slovaca* cases were found in *D. marginatus* ticks and only one in *D. reticulatus*, indicating a significant association between *R. slovaca* and *D. marginatus* (*p* < 0.0289).

**Figure 4:**
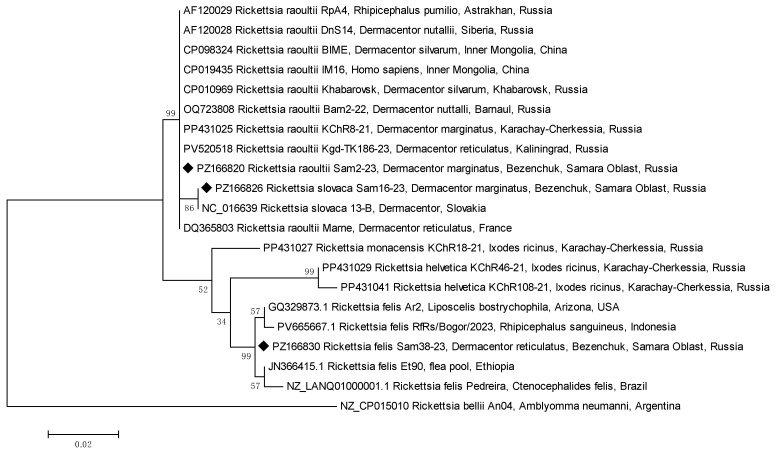
Phylogenetic tree constructed using the maximum likelihood method based on nucleotide sequences of *Rickettsia* spp. from ticks, including three from this study (Samara Oblast, black diamonds, ♦) and reference sequences of the *gltA* gene fragment (384 bp). The *R. bellii* An04 (NZ_CP015010) sequence was used as an outgroup. The GenBank accession numbers for reference sequences are shown with the sequence name, tick species, and country. The branch numbers indicate bootstrap support (1000 replicates). The scale bar indicates phylogenetic distance.

Because the *gltA* gene fragment sequences of the *R. raoultii* and *R. slovaca* samples were identical within each species, Figure 4 compares representative samples against related strains from the NCBI GenBank nr/nt database. *R. raoultii* samples exhibited 100% identity to all reference strains, including Khabarovsk, Marne, BIME, and IM16, as well as to our previously described isolates from Altai Krai, Karachay-Cherkessia, and Kaliningrad Oblast [7,8,9]. Similarly, *R. slovaca* was 100% identical to the Slovakian reference strain 13-B and previously reported samples from Karachay-Cherkessia [8].

**Figure 5:**
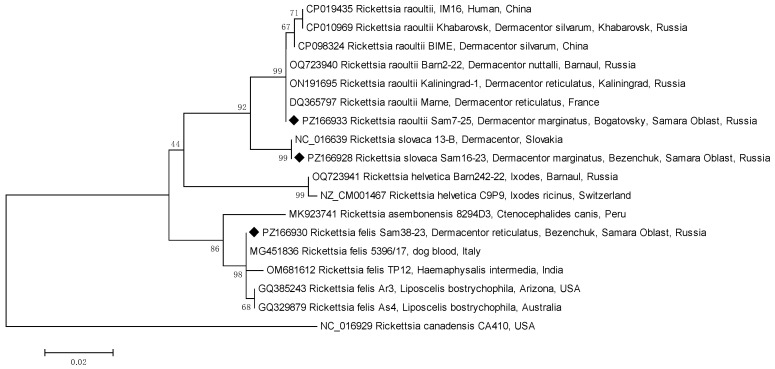
Phylogenetic tree constructed using the maximum likelihood method based on nucleotide sequences of *Rickettsia* spp. from ticks, including three from this study (Samara Oblast, black diamonds, ♦) and reference sequences of the *ompB* gene fragment (426 bp). The *R. canadensis* CA410 (NC_016929) sequence was used as an outgroup. The GenBank accession numbers for reference sequences are shown with the sequence name, tick species, and country. The branch numbers indicate bootstrap support (1000 replicates). The scale bar indicates phylogenetic distance.

In contrast, analysis of the *ompB* gene fragment revealed that the *R. raoultii* samples showed 97.42% identity to the Khabarovsk and IM16 reference strains, and 97.66% to the BIME strain (Figure 5). However, they were identical to the Marne strain from France and samples from Altai Krai and Kaliningrad Oblast, confirming their classification within the RpA4 genotype. Interestingly, two of the six *ompB*-sequenced *R. raoultii* samples from Kinelsky and Bogatovsky Districts differed from the other four by a 9-bp deletion (5′-ACTCCTGAA-3′). This deletion was also aligned with samples from Romania (JX683120), Italy (KJ663752), and Russia (ON515506, ON515508), suggesting that at least two different *R. raoultii* putative genotypes persist in the Samara Oblast. The *R. slovaca* samples remained 100% identical to the reference strain 13-B.

No coinfections of *R. raoultii* and *R. slovaca* were detected in the analyzed samples. Whether this indicates ecological exclusion [15], stochastic effects, or sampling limitations remains unclear.

No coinfections were detected in the samples analyzed. Whether this indicates ecological exclusion [15], stochastic effects, or sampling limitations remains unclear.

### 3.3. Identification of a Rickettsia Felis in Dermacentor Reticulatus

During sequencing of all SFGR-positive tick samples from the Bezenchuksky District, a single sample from *D. reticulatus* differed dramatically in its *gltA* gene from the expected sequences of *R. raoultii*/*R. slovaca* sympatric pattern. Out of the six gene fragments tested (*gltA*, *ompA*, *ompB*, *htrA*, *rrs*, and *sca4*), only two (*gltA* and *ompB*) were successfully amplified via PCR under standard conditions. Both sequences obtained shared the highest identity with *R. felis* and clustered within its clade on the phylogenetic trees (Figure 4 and Figure 5). Specifically, the *gltA* fragment demonstrated 99.74% sequence identity to the closest relative isolates: *R. felis* Et90 (JN366415) from a flea pool in Ethiopia and *R. felis* Ar2 (GQ329873) from a booklouse in the USA (Figure 4). The partial *ompB* sequence exhibited 100% identity with *R. felis* 5396/17 (MG451836) isolated from canine blood in Italy, and 99.3% identity to *R. felis* Ar3 (GQ385243) from a US booklouse (Figure 5).

To validate the morphological identification of *D. reticulatus*, we sequenced a fragment of the mitochondrial *cox1* gene from the tick sample that tested PCR-positive for *R. felis* (Figure 3). The molecular data confirmed the initial morphological identification. This confirms that the discovered *R. felis* was detected in *D. reticulatus*. To our knowledge, this represents the first molecular detection of *R. felis*-like DNA in *D. reticulatus* in Russia. Further studies are required to find out whether this species could be a biological vector or whether the finding is an incidental acquisition.

## 4. Discussion

In this study, we investigated the prevalence and species diversity of SFGR in *Dermacentor* ticks collected from Samara Oblast, a previously unexplored region in the Middle Volga region of Russia. Our main findings reveal a high overall prevalence of SFGR (33.5%), with *R. raoultii* as the dominant species (94.4% of identified cases), alongside a minor but consistent presence of *R. slovaca* (4.6%). This pattern confirms that Samara Oblast is a stable sympatric area for *D. reticulatus* and *D. marginatus* and extends the known epidemiological relevance of *Dermacentor*-associated rickettsiae in the Volga Federal District. Most notably, we report the first detection of *R. felis* in Russia and, globally, the first identification of this species in *D. reticulatus*.

Samara Oblast is one of the regions in Southeastern European Russia where the ranges of *D. reticulatus* and *D. marginatus* overlap. This overlap is driven by the ecological gradient from humid, meadow-like microhabitats typical of *D. reticulatus* to the more arid, steppe-like conditions preferred by *D. marginatus* [2,6]. The overall SFGR prevalence in *Dermacentor* ticks from Samara Oblast (33.5%) was comparable to or even higher than infection rates reported in other European and European Russian regions. For instance, studies in Slovakia reported a prevalence of 27.96% in *D. marginatus* and 28.76% in *D. reticulatus* [4], while in Kaliningrad Oblast, we previously found a prevalence of 6.1% in *D. reticulatus* [7]. Overall, in Europe, the prevalence of rickettsiae in *Dermacentor* ticks depends on the country, region, season, and year, and may vary widely from 0% up to 97% [16,17]. Interestingly, we observed a significantly higher infection rate in *D. marginatus* (44.7%) compared to *D. reticulatus* (27.5%) (*p* < 0.0001) This differential prevalence may reflect species-specific ecological preferences or host-seeking behavior, or variations in reservoir host availability. Additionally, climatic factors such as temperature and humidity influence tick activity, survival, and pathogen transmission dynamics. The continental climate of Samara Oblast, characterized by cold winters and warm summers, likely shapes the seasonal activity patterns of both tick species and may affect rickettsial prevalence through its impact on tick population dynamics and questing behavior. Future studies incorporating detailed microhabitat characterization and climate data would help elucidate the environmental drivers of SFGR prevalence in this region.

Parola et al. [3] hypothesized that *R. raoultii* is generally more prevalent than *R. slovaca* in *D. marginatus* populations across Europe. Later, however, it was demonstrated that the prevalence of *R. slovaca* was higher in *D. marginatus* (20.59–24.26%) than in *D. reticulatus* (1.69–3.43%) [4]. Furthermore, in Tuscany, Italy [18] and the Central Anatolia region of Turkey [19], *R. slovaca* was clearly dominant in *D. marginatus*, contrasting with Parola’s previous observations. Our findings align well with these studies, showing that *R. raoultii* was more prevalent overall in both *Dermacentor* species, whereas *R. slovaca* dominated in *D. marginatus* but occurred at significantly lower levels in *D. reticulatus* (*p* = 0.0289). Moreover, our previous data from Karachay-Cherkessia in the North Caucasus confirmed a similar pattern, with an overall *R. slovaca* prevalence of 2.5% to 5.7% in *D. marginatus* [8]. The predominance of *R. raoultii* in local tick populations suggests that this species may be epidemiologically important in the region; however, human clinical studies are needed to demonstrate its contribution to the disease.

When comparing SFGR prevalence across adjacent territories, relevant benchmarks can be drawn from Kazakhstan. Specifically, pooled *D. marginatus* and *D. reticulatus* samples from this country exhibited comparable minimal infection rates (MIR) of 21.6% and 21.4%, respectively. In alignment with our findings, among 122 *D. marginatus* pools analyzed there, 119 (97.5%) tested positive for *R. raoultii*, whereas a mere 3 (2.5%) tested positive for *R. slovaca*; furthermore, all 3 *D. reticulatus* pools were positive exclusively for *R. raoultii* [20]. Meanwhile, surveillance data from the Saratov Oblast revealed substantially higher MIRs in pooled *D. marginatus* and *D. reticulatus*: 79.6% (95% CI: 72.2–85.8%) and 47.4% (95% CI: 37.9–56.9%), respectively, further substantiating our observation that SFGR prevalence is more pronounced in *D. marginatus* than in *D. reticulatus* [21].

Additionally, the potential geographic range of *R. slovaca* may extend to *Dermacentor silvarum* and *Dermacentor nuttalli* within Asian Russia, given that this pathogen has been documented in these specific vector species in China [22,23]. Nevertheless, prior surveys of these tick species in Barnaul (Altai Krai, Western Siberia) [7] and the Russian Far East (Amur Oblast and Khabarovsk Krai) [24] detected only *R. raoultii*.

Phylogenetic analysis of *gltA* and *ompB* gene fragments revealed that *R. raoultii* strains from Samara Oblast are identical to the Marne strain from France and to strains previously identified by our group in Altai Krai and Kaliningrad Oblast [7,9]. However, partial sequencing of *ompB* identified two distinct *R. raoultii* putative genotypes circulating in the region: one with a 9-bp deletion and one without. This deletion has been observed in strains from Romania, Italy, and other parts of Russia, suggesting a wide geographic distribution of this genetic variant. The biological and clinical significance of this deletion remains unknown, but it may serve as a useful molecular marker for tracking strain dynamics and dispersal.

No coinfections of different SFGR species within a single tick were observed, consistent with the hypothesis of mutual exclusion, by which prior rickettsial infection may prevent transovarial or transstadial transmission of a second rickettsial species [15]. This phenomenon, often referred to as “rickettsial interference”, has been observed experimentally and may contribute to the mutual exclusivity of different SFGR species at the individual tick level, despite their regional sympatry within the same ecosystems. Nevertheless, there are some documented cases where SFG Rickettsia and Rickettsia from the typhus (TG) or transitional (TRG) groups can persist simultaneously within a single tick [25]. Importantly, sampling limitations, including the relatively small number of sequenced samples, may have prevented the detection of very rare coinfection events. The potential influence of stochastic effects should also be considered.

The most unexpected and striking finding of this study is the sporadic detection of *R. felis* in a single *D. reticulatus* tick from the Bezenchuksky District. *R. felis* is an emerging pathogen primarily associated with cat fleas (*Ctenocephalides felis*) and has been implicated in human rickettsioses worldwide, often presenting as flea-borne spotted fever (FBSF) [26,27]. Clinical manifestations typically include fever, rash, headache, myalgia, and an eschar at the bite site [28]. To our knowledge, this is the first report of *R. felis* in Russia and the first documented molecular detection of this species in *D. reticulatus* globally. However, *R. felis* has also been found in other flea species, mosquitoes [29], and has occasionally been detected in a few other tick species, including *Amblyomma maculatum* [30], *Amblyomma humerale* [31], *Rhipicephalus turanicus* [32], *Rhipicephalus bursa* [33], *Rhipicephalus sanguineus* [34,35], *Ixodes ricinus* [32,36], and *Ixodes hexagonus* [32]. Within the genus *Dermacentor*, *Dermacentor variabilis* [37] and *Dermacentor albipictus* [38] in the USA were recently added to the list of *R. felis* tick vectors. Our molecular identification of the pathogen in *D. reticulatus* was robust: the *gltA* and *ompB* sequences showed 99.74% and 100% identity to the reference *R. felis* strains, respectively, and subsequent tick species confirmation via *cox1* sequencing excluded any misidentification. The failure to amplify the remaining four loci (ompA, htrA, rrs, sca4) under standard PCR conditions likely reflects primer mismatches, low DNA quantity, DNA degradation, or stochastic amplification failure. This divergence is entirely consistent with the classification of R. felis within the transitional group (TRG) rather than the typical SFG. The unique genomic architecture of TRG rickettsiae separates them from classical SFGR, which underscores why universal primers may fail. Further high-throughput sequencing or genomic characterization is required to precisely delineate these target boundaries. This is further supported by our successful amplification of the gltA and ompB, which feature higher sequence conservation across both groups. Because *D. reticulatus* frequently feeds on domestic dogs and wild carnivores, which act as common hosts for fleas, this tick likely acquired the pathogen horizontally while co-feeding alongside *R. felis*-infected fleas on a shared mammalian host. Whether *D. reticulatus* can serve as a competent vector capable of biological transmission or if this represents an isolated dead-end spillover event requires further vector competence studies. Furthermore, the prospective field studies in this region will shed light on the distribution of this pathogenic rickettsial species in *Dermacentor* ticks.

We emphasize that molecular detection of *R. felis* DNA in a field-collected tick does not establish vector competence, nor does it indicate active infection of the tick. Distinguishing between DNA detection, infection, reservoir status, and biological transmission requires experimental transmission studies and further characterization.

The high overall SFGR prevalence (33.5%) in *Dermacentor* ticks from Samara Oblast indicates that residents and visitors to this region are at substantial risk of exposure to tick-borne rickettsiae. Clinicians should be aware of this entity when evaluating patients with scalp eschars and cervical lymphadenopathy following tick bites. The absence of other tick-borne pathogens in our samples suggests that SFGR may be the predominant tick-borne pathogen group in *Dermacentor* ticks in this region, although this does not exclude the presence of these pathogens in other tick species or at different sampling times. Finally, our findings highlight the need for enhanced surveillance, including year-round tick collection, analysis of additional tick species, and integration with human case data to better characterize the true public health burden of rickettsial infections in Samara Oblast.

This study has several limitations. First, the number of sequenced samples (47.4% of SFGR-positive ticks) was restricted, and targeted sequencing likely overestimates the proportion of *R. slovaca* because we sequenced all positive samples from districts where *R. slovaca* was initially detected. Second, the detection of *R. felis* in a single tick limits our ability to assess its prevalence, seasonality, and ecological persistence. Third, we did not perform whole-genome sequencing of the *R. felis* strain, since the culturing of this intracellular pathogen is technically challenging. Fourth, we did not assess the viability of the detected *R. felis* or confirm active infection in the tick. The presence of bacterial DNA does not necessarily indicate a replicative infection or the ability of the tick to transmit the pathogen. Vector competence studies are required to determine whether *D. reticulatus* can biologically transmit *R. felis*.

## 5. Conclusions

In summary, this study provides the first comprehensive baseline assessment of SFGR in *Dermacentor* ticks from Samara Oblast, demonstrating a high overall prevalence of *R. raoultii*, a minor but consistent presence of *R. slovaca*, and a strong association of the latter with *D. marginatus*. Crucially, the molecular detection of *R. felis* in *D. reticulatus* suggests a potential association that requires confirmation through additional studies and represents the first documented report of *R. felis* in Russia. While the public health significance of this finding remains to be fully elucidated, it underscores the critical importance of continued epidemiological surveillance for unexpected rickettsial species in local tick populations. Our data indicates that clinicians should remain vigilant, as patients presenting with tick-borne lymphadenopathy (TIBOLA/DEBONEL/SENLAT) may emerge within Samara Oblast. Future research should focus on vector competence studies for *R. felis* in *D. reticulatus*, longitudinal surveillance to assess temporal trends in prevalence, and integration with human clinical data to better define the disease burden in this region.

## Figures and Tables

**Table 1:** Prevalence of SFGR in *Dermacentor* ticks from Samara Oblast, 2023–2025.

Tick Species	Number of Ticks	SFGR Positive, n (%, 95% CI %)	Number of *gltA* Sequenced, n (%)	*R. raoultii*, n (%)	*R. slovaca*, n (%)	*R. felis*, n (%)
*D. marginatus*	237	106 (44.7, 38.5–51.1)	36 (34.0)	32 (88.9)	4 (11.1)	0
*D. reticulatus*	444	122 (27.5, 23.5–31.8)	72 (59.0)	70 (97.2)	1 (1.4)	1 (1.4)
Total	681	228 (33.5, 30.0–37.1)	108 (47.4)	102 (94.4)	5 (4.6)	1 (1.0)

**Table 2:** Prevalence of SFGR in *Dermacentor* ticks from 15 districts of Samara Oblast, 2023–2025.

District, Settlement	Year, Month, Day	Coordinates	Tick Species	Number of Ticks	SFGR Positive, n (%, 95% CI %)	*gltA* Sequenced, n (%)
Bezenchuksky, Bezenchuk	20 April 2023	52.99547°	*D. reticulatus*	20	3 (15.0, 4.4–36.9)	3 (100.0) ^†^
		49.34595°	*D. marginatus*	20	8 (40.0, 21.8–61.4)	8 (100.0) ^‡^
Krasnoyarsky, Khoroshenkoe	5 April 2023	53.535622°	*D. reticulatus*	10	3 (30.0, 10.3–60.8)	3 (100.0)
		50.639146°	*D. marginatus*	30	13 (43.3, 27.4–60.8)	3 (23.1)
Kinelsky, Chubovka	15 May 2023	53.387456°	*D. reticulatus*	30	13 (43.3, 27.4–60.8)	13 (100.0) ^§^
		50.552217°	*D. marginatus*	10	3 (30.0, 10.3–60.8)	3 (100.0)
Volzhsky, Nikolaevka	1 April 2023	53.088694°	*D. reticulatus*	20	12 (60.0, 38.6–78.2)	3 (25.0)
		50.322036°	*D. marginatus*	20	13 (65.5, 43.7–82.0)	3 (23.1)
Volzhsky, Chernoreche	6 April 2023	53.116627°	*D. reticulatus*	23	4 (17.4, 6.4–37.7)	3 (75.0)
		50.263923°	*D. marginatus*	17	10 (58.8, 35.9–78.4)	3 (30.0)
Pestravsky, Vysokoe	23 April 2024	52.393808°	*D. reticulatus*	9	2 (22.2, 5.3–55.7)	2 (100.0)
		50.031938°	*D. marginatus*	46	20 (43.5, 30.2–57.8)	3 (15.0)
Krasnoarmeyskiy, Leninskii	23 April 2024	52.740814°	*D. marginatus*	50	21 (42.0, 29.4–55.8)	3 (14.3)
		50.153635°				
Alekseyevskiy, Antonovka	2 May 2024	52.671961°	*D. reticulatus*	15	5 (33.3, 15.0–58.5)	3 (60.0)
		51.222846°	*D. marginatus*	35	12 (34.3, 20.8–50.9)	3 (25.0)
Neftegorsky, Barinovka	2 May 2024	52.862776°	*D. reticulatus*	100	19 (19.0, 12.4–27.9)	3 (15.8)
		50.825358°				
Pokhvistnevsky, Staryi Amanak	13 May 2024	53.724343°	*D. reticulatus*	50	14 (28.0, 17.4–41.8)	3 (21.4)
		51.899856°				
Bogatovsky, Arzamastsevka	2 April 2025	53.168409°	*D. reticulatus*	23	18 (78.3, 57.7–90.8)	17 (94.4)
		51.39804°	*D. marginatus*	7	5 (71.4, 35.2–92.4)	5 (100.0) ^§^
Shigonsky, Shigony	3 April 2025	53.422795°	*D. reticulatus*	30	2 (6.7, 0.8–22.4)	2 (100.0)
		48.779978°				
Stavropolsky, Bakhilovo	7 April 2025	53.379541°	*D. reticulatus*	30	7 (23.3, 11.5–41.2)	3 (42.9)
		49.644483°				
Shentalinsky, Kamenka	15 April 2025	54.475471°	*D. reticulatus*	29	8 (27.6, 11.3–43.8)	8 (100.0)
		51.937411°	*D. marginatus*	1	1 (100.0, 16.7–100.0)	1 (100.0) ^§^
Isaklinsky, Dva Kliucha	15 April 2025	54.093527°	*D. reticulatus*	30	6 (20.0, 9.1–37.7)	3 (50.0)
		51.541368°				
Chelno-Vershinsky, Krasnyi Stroitel	15 April 2025	54.237913°	*D. reticulatus*	24	5 (20.8, 8.8–40.9)	3 (60.0)
		51.157475°	*D. marginatus*	2	1 (50.0, 9.5–90.5)	1 (100.0)
Total	2023–2025			681	228 (33.5, 30.0–37.1)	108 (47.4)

All SFGR identified as *R. raoultii*, except for: ^†^ *R. felis* (one sample); ^‡^ *R. slovaca* (two samples); ^§^ *R. slovaca* (one sample).

## Data Availability

The sequences from this study are available in the NCBI GenBank under accession numbers PZ166815-PZ166934, PZ436268-PZ436272 and PZ448574-PZ448579.

## Supplementary Materials

The following supporting information can be downloaded at https://www.mdpi.com/article/10.3390/microorganisms14071461/s1. Table S1: Results of pilot study for prevalence of SFGR in *Dermacentor* spp. tick pools from the Samara Oblast, 2022.

## Abbreviations

The following abbreviations are used in this manuscript:

SFGR	Spotted fever group *Rickettsia*
TBD	Tick-borne disease
TBP	Tick-borne pathogen
TIBOLA	Tick-borne lymphadenopathy
DEBONEL	*Dermacentor*-borne necrosis erythema and lymphadenopathy
SENLAT	Scalp eschar and neck lymphadenopathy after tick bite
MSF	Mediterranean spotted fever
NATT	North Asian tick typhus
ASF	Astrakhan spotted fever
CRIE	Central Research Institute of Epidemiology
qPCR	Quantitative polymerase chain reaction
PCR	Polymerase chain reaction
TBEV	Tick-borne encephalitis virus
rRNA	Ribosomal ribonucleic acid
kDa	Kilodalton
CI	Confidence interval
TG	Typhus group
TRG	Transitional group
FBSF	Flea-borne spotted fever
MIR	Minimal infection rate
