# Supplementary material for: Sympatric Occurrence of Rickettsia slovaca and Rickettsia raoultii in Dermacentor Ticks from Samara Oblast and the First Molecular Detection of Rickettsia felis in Russia and Globally in Dermacentor reticulatus
Source: Microorganisms. 2026 Jul 2;14(7):1461. doi: 10.3390/microorganisms14071461 (PMC13413530; doi:10.3390/microorganisms14071461)
Supplement: Supplementary file 1 [file microorganisms-14-01461-s001.zip › microorganisms-4382174-supplementary.pdf]

Table S1. Results of pilot study for prevalence of SFGR in *Dermacentor* spp. tick pools from the Samara Oblast, 2022.

District, Settlement	Year, Month, Day	Coordinates	Number of Ticks	Genospecies Found
Kinelsky, Padovka	2022 May 1	52.465833°, 49.513333°	111	*R. slovaca*
Volzhsky, Kurumoch	2022 May 2	53.488889°, 50.037222°	14	*R. raoultii*
Krasnoyarsky, Khoroshenkoe	2022 May 6	53.543611°, 50.596111°	45	*R. raoultii*
Krasnoyarsky, Khoroshenkoe	2022 May 22	53.535622°, 50.639146°	55	*R. slovaca*
